# The reduced bactericidal activity of neutrophils as an incisive indicator of water-immersion restraint stress and impaired exercise performance in mice

**DOI:** 10.1038/s41598-019-41077-5

**Published:** 2019-03-14

**Authors:** Manabu Kinoshita, Hiroyuki Nakashima, Masahiro Nakashima, Minori Koga, Hiroyuki Toda, Kazuki Koiwai, Yuji Morimoto, Hiromi Miyazaki, Daizoh Saitoh, Hiroaki Suzuki, Shuhji Seki

**Affiliations:** 1 Department of Immunology and Microbiology, National Defense Medical College, Namiki 3-2, Tokorozawa, 359-8513 Japan; 2 Department of Psychiatry, National Defense Medical College, Namiki 3-2, Tokorozawa, 359-8513 Japan; 3 Department of Physiology, National Defense Medical College, Namiki 3-2, Tokorozawa, 359-8513 Japan; 4 Division of Traumatology, Research Institute, National Defense Medical College, 3-2 Namiki, Tokorozawa, 359-8513 Japan; 5 Graduate School of Pure and Applied Sciences, University of Tsukuba, 1-1-1 Tennodai, Tsukuba, Ibaraki 305-8573 Japan

## Abstract

The incisive evaluation of psychological stress may be required to determine the exercise performance of stressed hosts. We investigated objective markers of psychological stress that reflect exercise performance, focusing on the neutrophil function. We used murine water-immersion restraint (WIR) stress for our assessments. After receiving WIR for 1 or 2 h, mice were exercised on an airtight treadmill that monitors their respiratory exchange ratio. The neutrophil function was analyzed after WIR stress. Although the control mice (without WIR) showed good combustion of both carbohydrates and lipids as energy sources during treadmill exercise, mice that underwent 2-h WIR did not combust carbohydrates or lipids effectively, drastically reducing their performance. In contrast, the 1-h WIR mice showed carbohydrate combustion (albeit a slow response) but did not use lipids for energy, thereby running longer than the 2-h WIR mice but shorter than the control mice. The bactericidal activity of neutrophils, but not their superoxide production or microsphere-phagocytic activity, was significantly reduced by 1-h WIR and further reduced by 2-h WIR, indicating a significant association between WIR stress and exercise performance. The neutrophil bactericidal activity may be a good indicator of psychological stress and a useful tool for precisely assessing exercise performance.

## Introduction

Psychological stress has a negative impact on a wide range of physical health outcomes. It has been implicated in the pathogenesis of coronary disease^1^ and the incidence of acute myocardial infarction^2^. Psychological stress also may be involved in vulnerability of the host defense^3^ and associated with excessive fatigue^4,5^. It has also been reported that the measurement of psychological stress may become an important variable in the analysis of individual training responses after exercise interventions, because the mental stress, which was self-rated, affected the response of healthy volunteers to exercise training^6^. Although the severity may differ among athletes, most of them experience a certain degree of psychological stress at important events, such as the Olympics^7,8^. Thus, the precise evaluation of psychological stress in athletes may be important for allowing them to be in their best condition at such events and to elicit their best exercise performance.

Nevertheless, it is quite difficult to objectively and precisely evaluate psychological stress. Stress hormones such as cortisol are considered to be representative stress markers; however, these hormones show circadian variation^9^. Reactive oxygen species (ROS) produced from neutrophils are reportedly involved in psychological stress, especially in cases of stress-induced gastric ulcer that are produced by water-immersion restraint (WIR) in mice or rats^10,11^. Although increasing stress hormones or ROS production reportedly indicates the severity of psychological stress, they are basically produced to protect the hosts from harmful stimuli. For example, the ROS evoked immediately after burn injury are crucial for the host defense to restore the neutrophil function^12^. In addition, ROS generation sometimes shows a paradoxical response in hosts. We focused on the neutrophil bactericidal activity in the present study. We believe that bactericidal activity is a reliable indicator of the host defense activity. Increased bactericidal activity is beneficial for hosts, while decreased activity is definitely harmful. Interestingly, psychological stress reportedly reduced the bactericidal activity of neutrophils in children^13^.

An incisive and objective marker of psychological stress that reflects exercise performance would be quite useful for athlete conditioning. We therefore investigated how psychological stress affects exercise performance and the bactericidal activity of neutrophils, using a murine WIR stress model, which is commonly used as a psychological stress model, although it could not completely exclude the aspect of physiological (albeit non-exercise) stress. Mice and other rodents are considered social animals; therefore, the disruption of social hierarchies can lead to psychological stress^14^. The destruction of existing social structures may induce psychological stress without adding any physiological stress. We hope to use such murine models in future studies. If psychological stress can be precisely evaluated based on the neutrophil bactericidal activity, it may be a useful tool for effectively eliciting exercise performance in hosts.

## Results

### The effect of WIR stress on murine exercise performance

**Figure 1:**
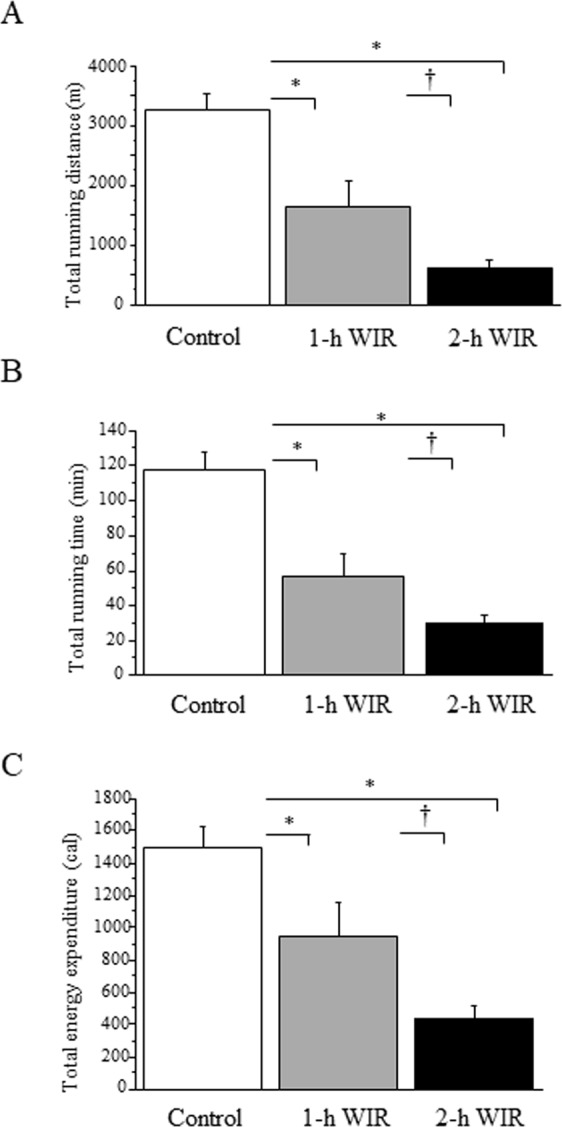
The treadmill performance of WIR mice. The mice in the 1-h WIR, 2-h WIR, and control groups were run on the treadmill until exhaustion. Their running distance (**A**), duration (**B**), and total energy expenditure (**C**) were examined. The data are shown as the mean ± SE from 15 mice in the 2-h WIR group, 10 mice in the 1-h WIR group, and 50 control mice. A standard one-way ANOVA followed by the Bonferroni post-hoc test was performed. *Indicates p < 0.01, and ^†^indicates p < 0.05.

Mice that underwent WIR stress for 1 or 2 h showed drastically reduced performance in treadmill exercise, as assessed by running distance and duration, in comparison to the control mice (Fig. 1A,B). The exercise performance of the 2-h WIR group more severely impaired in comparison to the 1-h WIR group (Fig. 1A,B). Total energy expenditure (EE) during treadmill exercise until exhaustion was also markedly decreased in the 1-h WIR group and was further reduced in the 2-h WIR group (Fig. 1C). WIR stress may severely impair murine exercise performance and reduce their consumable energy. The weight loss before and after 1-h or 2-h of WIR stress did not differ to a statistically significant extent (Table 1).

**Table 1:** Changes in mouse body weight and neutrophil counts after WIR stress.

Variable	1 h-WIR mice		2 h-WIR mice	
	Before	After	Before	After
Body weight (g)	24.4 ± 0.3	24.2 ± 0.3	24.2 ± 0.2	23.8 ± 0.2
Neutrophil counts (×10^3^/μl)	2.9 ± 0.5	2.9 ± 0.5	2.8 ± 0.4	1.8 ± 0.3*

Mice received WIR stress for 1 or 2 h. Their body weights and neutrophil counts were measured before and after WIR stress. Data were shown mean ± SE from 11 of 1 h-WIR mice, and 13 of 2 h-WIR mice. A paired Student’s *t*-test was performed before and after WIR in the 1- and 2-h WIR mouse groups. *p < 0.01 vs. before WIR.

### The effects of WIR stress on oxygen (O_2_) consumption and carbon dioxide (CO_2_) production in mice during treadmill exercising

**Figure 2:**
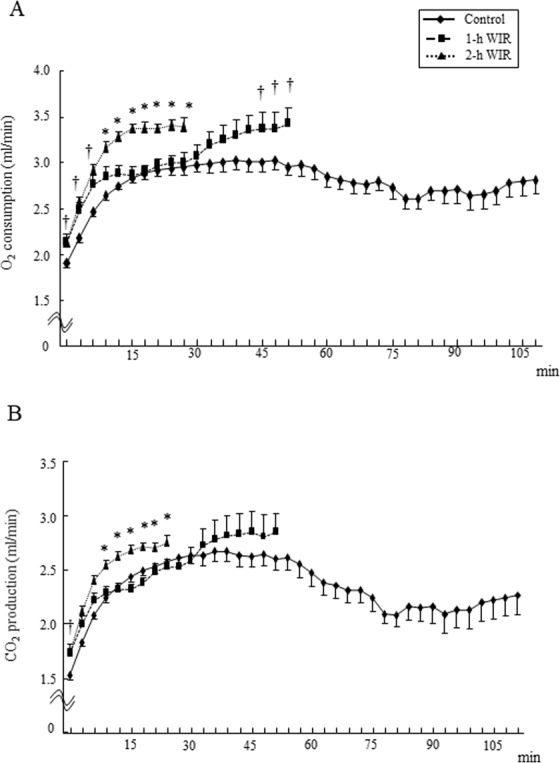
The O_2_ consumption and CO_2_ production during treadmill exercise in WIR mice. The mice in the 1-h WIR, 2-h WIR, and control groups were run on the treadmill and their O_2_ consumption (**A**) and CO_2_ production (**B**) were monitored. The data are shown as the mean ± SE from 15 mice in the 2-h WIR group, 10 mice in the 1-h WIR group, and 50 control mice. A standard one-way ANOVA followed by the Bonferroni post-hoc test was performed at each time point. *Indicates p < 0.01 vs. Control and p < 0.05 vs. 1-h WIR, and ^†^indicates p < 0.05 vs. Control.

The mice in both the 1-h and 2-h WIR groups showed significantly higher O_2_ consumption before treadmill running than the control mice and the O_2_ consumption was subsequently increased until 9 min after the start of running (Fig. 2A). The O_2_ consumption of the 2-h WIR group continuously increased beyond 12 min and the mice gave up running at approximately 30 min due to exhaustion (Fig. 2A). In contrast, the increase in O_2_ consumption at 12–30 min was suppressed in the mice in the 1-h WIR group but eventually increased beyond 30 min and the mice gave up running at approximately 50 min (Fig. 2A). Control mice were able to run for more than 100 min without a marked increase in O_2_ consumption (>3 ml/min) (Fig. 2A). The mice in the 2-h WIR group also showed significantly higher CO_2_ production beyond 12 min after the start of running in comparison to the 1-h WIR and control groups; they then gave up running (Fig. 2B). The mice in the 1-h WIR group tended to show increased CO_2_ production beyond 45 min in comparison to the control mice and then gave up running (Fig. 2B).

### The effect of WIR stress on the respiratory exchange ratio (RER) and energy expenditure (EE) during treadmill exercise in mice

**Figure 3:**
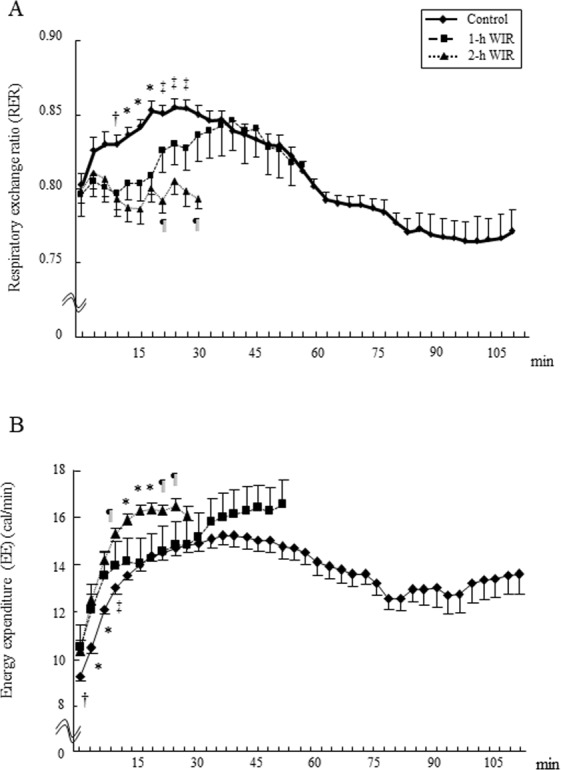
The respiratory exchange ratio (RER) and energy expenditure (EE) during treadmill exercise in WIR mice. The mice in the 1-h WIR, 2-h WIR, and control groups were run on the treadmill and their RER (**A**) and EE (**B**) were monitored. Data are shown as the mean ± SE from 15 mice in the 2-h WIR group, 10 mice in the 1-h WIR group, and 50 control mice. A standard one-way ANOVA followed by the Bonferroni post-hoc test was performed at each time point. *Indicates p < 0.01 vs. others, ^†^indicates p < 0.05 vs. others, ^‡^indicates p < 0.01 vs. 2-h WIR and p < 0.05 vs. 1-h WIR, and ^¶^indicates p < 0.01 vs. Control and p < 0.05 vs. 1-h WIR.

Control mice showed an increased RER in the initial phase of running (until 45 min) (Fig. 3A), suggesting that carbohydrates were predominantly used for exercise in the initial phase. In turn, their RER was decreased in the subsequent phase (beyond 60 min) (Fig. 3A), indicating lipid oxidation. In contrast, the mice in the 2-h WIR group did not show an increased RER until they gave up running. Interestingly, the RER of the mice in the 1-h WIR group did not increase until 30 min but gradually increased beyond 30 min. However, their RER did not decrease below 0.8 until they gave up running (Fig. 3A). The mice in both the 1-h and 2-h WIR groups showed an increased EE (per-minute expenditure) even before running and after the start of running (at 6–9 min) (Fig. 3B); however, their total consumable energy (total EE) was reduced (Fig. 1C). The 2-h WIR mice showed a markedly increased EE beyond 12 min and gave up running (Fig. 3B). The 1-h WIR mice did not show an increased EE until 30 min but it tended to be increased beyond 45 min and they gave up running (Fig. 3B).

### The effect of WIR stress on the superoxide production and phagocytic activity of neutrophils

**Figure 4:**
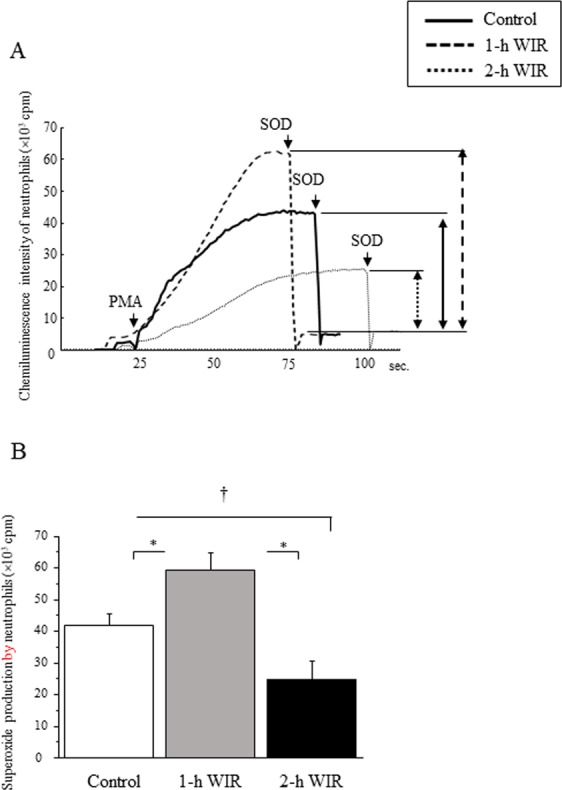
Superoxide production by neutrophils in WIR mice. Neutrophils were purified from the peripheral blood of mice in the 1-h WIR, 2-h WIR, and control groups to measure their MCLA-dependent chemiluminescence intensity following PMA stimulation. Representative data (**A**) and the superoxide production in each group (**B**) are shown. The data are shown as the mean ± SE from 6 mice in the 2-h WIR group, 11 mice in the 1-h WIR group, and 19 control mice. A standard one-way ANOVA followed by the Bonferroni post-hoc test was performed. *Indicates p < 0.01, and ^†^indicates p < 0.05.

**Figure 5:**
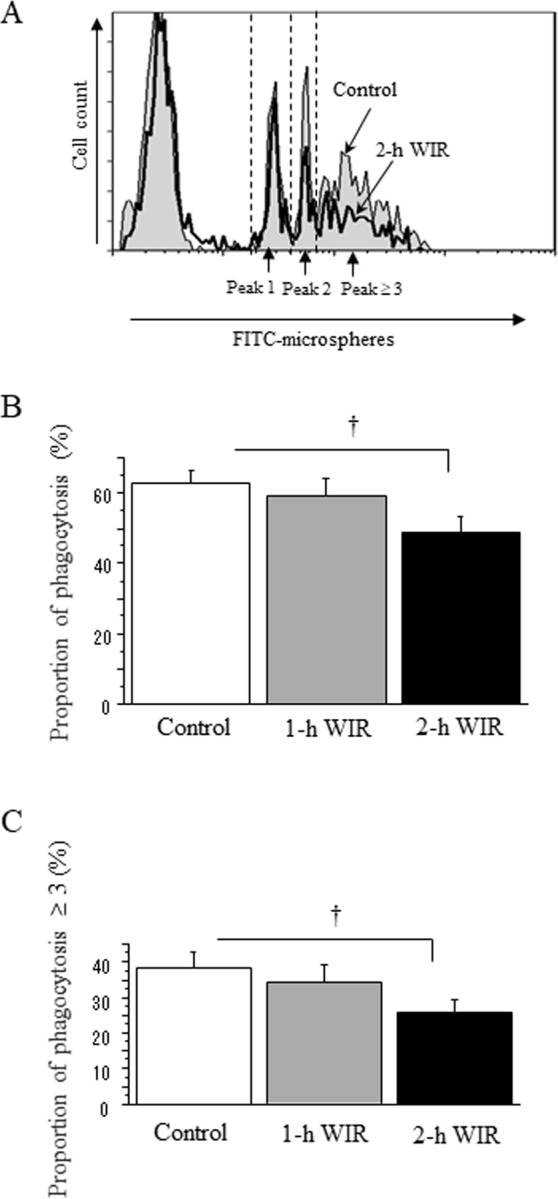
The phagocytic activity of neutrophils in WIR mice. Neutrophils were purified from the peripheral blood of mice in the 1-h WIR, 2-h WIR, and control groups and their microsphere-phagocytic activity was examined using flowcytometry. Representative data from mice in the 2-h WIR group and control mice are shown (**A**). The proportion of microsphere-phagocytosed neutrophils (peak 1, 2, and ≥3; **B**) and (peak ≥ 3; **C**) are shown. Data are shown as the mean ± SE from 24 mice in the 2-h WIR group, 21 mice in the 1-h WIR group, and 28 control mice. A standard one-way ANOVA followed by the Bonferroni post-hoc test was performed. ^†^Indicates p < 0.05.

Although 1-h WIR stress did not affect the neutrophil count in mice, 2-h WIR stress significantly reduced the neutrophil count (Table 1). Interestingly, the 2-methyl-6-(p-methoxyphenyl)-3,7-dihydroimidazo [1,2-a] pyrazine-3-one (MCLA)-dependent chemiluminescence intensity of the neutrophils was increased by phorbol 12-myristate 13-acetate (PMA) stimulation in the mice in the 1-h WIR group in comparison to that of the control mice, while it was reduced in the mice in the 2-h WIR group (Fig. 4A, representative data shown). Superoxide production, as assessed by the reduction of chemiluminescence intensity by superoxide dismutase (SOD), was significantly increased in the mice in the 1-h WIR group in comparison to in the control group but was decreased in the mice in the 2-h WIR group (Fig. 4B). The analysis of microsphere-phagocytosis revealed that the number of neutrophils with peaks of 2 and ≥3 was reduced in the 2-h WIR group in comparison to the control group (Fig. 5A, representative data shown). The proportion of neutrophils that phagocytosed microspheres (peaks 1, 2, and ≥3) in the mice in the 2-h WIR group was significantly decreased in comparison to the control group; a similar tendency was not observed in the 1-h WIR group (Fig. 5B). We further examined the proportion of neutrophils that phagocytosed more than three microspheres (peak ≥3), which was considered to represent potent phagocytic activity. The proportion was also reduced in the 2-h WIR group but not in the 1-h WIR group (Fig. 5C).

### The effect of WIR stress on neutrophil bactericidal activity and plasma cortisol levels in mice

**Figure 6:**
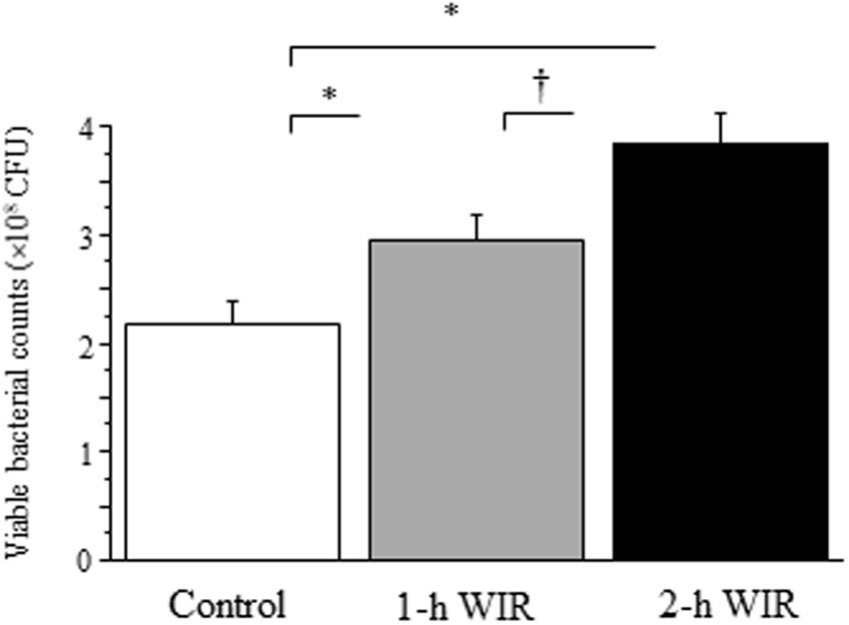
The bactericidal activity of neutrophils in WIR mice. Neutrophils were purified from the peripheral blood of mice in the 1-h WIR, 2-h WIR, and control groups and their *E. coli*-killing activity were examined. Data are shown as the mean ± SE from 11 mice in the 2-h WIR group, 11 mice in the 1-h WIR group, and 12 control mice. A standard one-way ANOVA followed by the Bonferroni post-hoc test was performed. *Indicates p < 0.01, and ^†^indicates p < 0.05.

**Figure 7:**
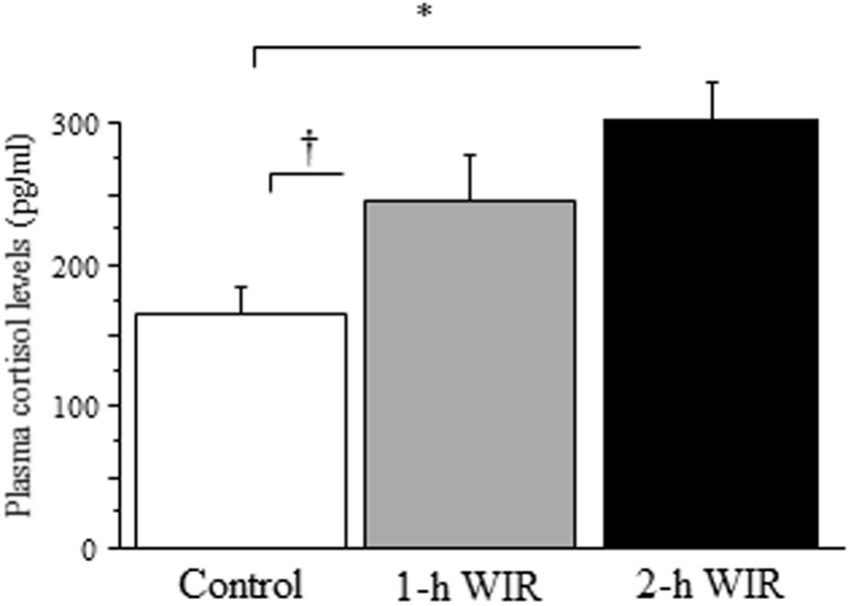
The plasma cortisol levels in WIR mice. Blood samples were obtained from the mice in the 1-h WIR, 2-h WIR, and control groups to measure the plasma cortisol levels. Data are shown as the mean ± SE from 21 mice in the 2-h WIR group, 11 mice in the 1-h WIR group, and 18 control mice. A standard one-way ANOVA followed by the Bonferroni post-hoc test was performed. *Indicates p < 0.01, and ^†^indicates p < 0.05.

The bacterial counts after co-incubation with neutrophils were significantly higher in both the 2-h and 1-h WIR groups than they were in the control group (Fig. 6), suggesting that the bactericidal activity of neutrophils was reduced in both WIR groups. Notably, 2-h WIR stress reduced the bactericidal activity further than 1-h WIR stress, suggesting that the bactericidal activity of neutrophils is strongly associated with the duration of WIR stress. The mice in both the 2-h and 1-h WIR showed markedly increased plasma cortisol levels; however, no statistically significant difference in these levels was observed between the two groups (Fig. 7).

## Discussion

The WIR model is widely used as a rodent model of psychological stress^10,11,15–21^. The duration of WIR stress was strongly related to murine exercise performance, because the treadmill performance of the mice in the 2-h WIR group was significantly reduced in comparison to the mice in the 1-h WIR group (Fig. 1A,B). Their O_2_ consumption and CO_2_ production were increased earlier during treadmill exercise in comparison to the mice in the 1-h WIR group (Fig. 2A,B). EE per minute in the mice in the 2-h WIR group was also increased earlier than in the mice in the 1-h WIR group (Fig. 3B), while their total consumable energy until exhaustion was markedly reduced in comparison to the 1-h WIR group (Fig. 1C), suggesting that mice in the 2-h WIR group could not effectively elicit energy for exercise.

The analysis of the RER revealed that the control mice combusted carbohydrates as energy in the early phase of treadmill exercise and subsequently used lipids for energy (Fig. 3A). This change in RER is similar to that of the athletes doing endurance sports, such as triathlon^22^. The process of carbohydrate oxidation to provide energy is more rapid than that of lipid oxidation. Because lipids require several more steps than carbohydrates to become acetyl CoA and enter the citric acid cycle, lipids make energy available more slowly in comparison to carbohydrates^23^. However, lipids can provide more energy than carbohydrates: 1 g of lipids can provide approximately 9 kilocalories while 1 g of carbohydrates provides only 4 kilocalories^23^.

The mice in the 2-h WIR group could not effectively use carbohydrates or lipids as energy, which might have caused the severe reduction of their exercise performance. In contrast, despite showing a slow response, the mice in the 1-h WIR group could use carbohydrates for energy, but they could not use lipids. Thus, the mice in the 1-h WIR group may be able to run longer on a treadmill than the mice in the 2-h WIR group but for less time than the control mice due to incomplete lipid combustion. The 2-h WIR may damage mice more severely than 1 h-WIR.

Metabolic disorders, such as the incomplete combustion of carbohydrates and/or lipids, renders the hosts energy deficient, resulting in attenuation of host defense activities^23^. Burn injuries, which are among the most severe forms of surgical stress, cause stress-induced hyperglycemia and an increased EE in patients, resulting in metabolic disturbance^24,25^. They also increase the host’s susceptibility to bacterial infection, because the host defense including neutrophil function, such as bactericidal activity, are profoundly impaired by the burn injury^26,27^. Metabolic disorder following surgical stress may be closely involved in the impairment of the host defense activity.

Neutrophil dysfunction was also observed in the murine WIR stress model. The WIR stress was strongly associated with the reduced bactericidal activity of neutrophils (Fig. 6). Two-hour WIR reduced the bactericidal activity further than 1-h WIR, suggesting that bactericidal activity may be a good indicator of WIR stress. In contrast, the neutrophil phagocytic activity, as assessed by microsphere-phagocytosis, may be not incisive enough to evaluate murine 1-h WIR stress (Fig. 5). Consistently, psychological stress in children reportedly reduced the bactericidal activity of neutrophils but not their phagocytic activity^13^.

The process of bacterial killing by neutrophils includes several steps other than phagocytosis. Superoxide production is one of the most important factors for neutrophil bacterial killing; however, the excessive production of superoxide conversely causes cell/tissue injuries. The increased production of superoxide by PMA-stimulated neutrophils induced by 1-h WIR stress may possibly cause cell/tissue damage, resulting in a reduction of bactericidal activity (Figs 4 and 6). PMA potently activates neutrophils via the activation of their protein kinase C^28^. PMA-stimulated superoxide production by neutrophils was also reportedly augmented under certain psychological stress conditions^29,30^, although there have been several reports that PMA-stimulated neutrophil superoxide production is conversely reduced under stress conditions^31,32^. Several investigators have reported that WIR stress causes excessive neutrophil activation to enhance their superoxide production, and that this may cause stress-induced gastric ulcers^10,11^. These investigators presumably focused on the harmful aspects of neutrophil-producing superoxide anions; however, they are basically indispensable for the host defense. Two-hour WIR stress may damage the neutrophils too severely to sustain their superoxide production; it also reduced neutrophil phagocytic activity (Fig. 5).

The neutrophil count in the peripheral blood was reduced under 2-h WIR stress (but not 1-h WIR stress) (Table 1), although it is often increased under conditions of psychological stress^11,33^. Neutrophils were seriously damaged by 2-h WIR stress and thereby presumably became moribund, destined to die or suffer apoptosis. Bone marrow typically expeditiously responds to such changes in neutrophils and increases their number in the circulation. However, in the current study, blood samples collected to count neutrophils were obtained from the mice immediately after 2-h WIR. We believe that the bone marrow was unable to adequately respond in such a short duration, resulting in the neutrophil counts not being restored or increased in the 2-h WIR mice.

Plasma cortisol levels may also be a good indicator of WIR stress. Nevertheless, we could not find a statistically significant difference in the cortisol levels between 1-h and 2 -h WIR groups. We collected blood samples at the same time (approximately 12:00) in all experiments in order to avoid the effect of circadian variation. Considering the clinical usage of cortisol as a psychological stress marker, this issue should be resolved.

In contrast, the bactericidal activity of neutrophils may be a useful tool for objectively assessing psychological stress. Consistently, psychological stress is also related to the susceptibility to the common cold^3^. We recently developed a microfluidic device coupled with a microfabricated oxygen electrode for the measurement of neutrophil bactericidal activity^34^. It can quickly measure the neutrophil bactericidal activity within several minutes. We intend to use this device to evaluate the neutrophil killing activity in mice subjected to WIR stress. In the near future, we would like to evaluate the psychological conditions of athletes through the measurement of neutrophil bactericidal activity using this device.

Spaceflight is also a unique model of psychological and physiological stress, as astronauts face substantial stress both psychologically and physiologically during their missions^33,35^. Dysregulation of the immune system, including neutrophil dysfunction, may occur during spaceflight, although limited precise in-flight data have been collected during long-duration spaceflight^33,35^. For exploration-class deep space missions, such as lunar or Mars missions, such immune dysregulation induced by spaceflight may become a serious concern for astronauts^35^. The development of new technology to diagnose immune changes in minute amounts of blood should therefore be encouraged^33^.

Several limitations associated with the present study warrant mention. WIR is one of the most commonly used stress paradigms, combining psychological stress and physical stress. However, we were unable to exclude physical factors (albeit non-exercise stress) as a cause of reduced exercise performance. We consider that it is difficult to exactly simulate human psychological stress using animal models, especially rodent models. Disruption of social hierarchies may induce psychological stress in animals without the need to administer physiological stress^14^. Further studies using such models should be performed in order to analyze the relationship between the psychological stress and exercise performance in greater detail, and we also should investigate the neutrophil bactericidal activity in various settings of human stress.

## Materials and Methods

### Animals

The Ethics Committee of Animal Care and Experimentation, National Defense Medical College Japan, approved all requests for animals and the intended procedures of the present study (permission No. 17040). All experiments were performed in accordance with relevant guidelines and regulations. A total of 268 C57BL/6 mice (male, 8-week-old, Japan SLC, Hamamatsu, Japan) were used. Mice were housed at 3~5 per cage (most cases were 5 per cage) under controlled temperature (23–25 °C) and relative humidity (50%) with 12 h of light (7:00–19:00). We used a 182 × 260 × 128-mm mouse polysulfone (PSF) cage (CL-0103-3; CLEA, Tokyo, Japan). These cages were set in an environmental control breeding system rack (KS-05W; Ishihala Co., Ltd., Tokyo, Japan), which is equipped with a high-efficiency particulate air (HEPA) filter (Cambridge Filter Japan, Ltd., Tokyo, Japan) on the rack top and an automatic water feeding system. We used Paper Clean (Japan SLC) as a breeding bed for mice and changed the bedding twice a week. Water was automatically supplied by the breeding system rack. They were fed a standard ustulation diet (NMF; Oriental Yeast Co. Tokyo, Japan) with *ad libitum* access to food and water during a 1-week adaptation period before experiments. All experiments were conducted between 9:00 and 13:00.

### WIR stress

Mice were exposed to WIR stress by immersion in water for 1 or 2 h at 37 °C using a vertical plastic restrainer (diameter, 30 mm; length, 80 mm) (ICN-2^®^, ICM Science Technology Development Co., Ltd., Tsukuba, Japan). The mice were immersed up to their shoulders and were considered immobilized when they ceased to struggle and their limb movements reached a minimum. They were in contact with the water during the WIR stress, as the plastic container used was not sealed against water. The control mice were moved from their home cages to new housing cages of the same type (CL-0103-3) and placed there for 2 h, as we wanted to compare the mice with and without WIR stress; therefore, we set both mouse groups in a new environment (WIR or no WIR new housing condition). Seventy-seven mice were used for 2-h WIR, 64 for 1-h WIR, and 127 as controls (without WIR). After WIR/sham stress, treadmill running was performed in 15 of the 2-h WIR mice, 10 of the 1-h WIR mice, and 50 control mice to analyze their exercise performance, O_2_ consumption, and CO_2_ production. Regarding the murine treadmill exercise following WIR/sham stress, we picked up one mouse from the home cage at a time and subjected it to 1- or 2-h WIR/sham stress. Immediately after WIR/sham stress, we ran it on the treadmill. After finishing treadmill exercise, we did not return this mouse to the home cage but set it in a new cage. This manipulated mouse was not used/examined anymore and was euthanized using isoflurane (Forane^®^; AbbVie GK, Tokyo, Japan). Neutrophils were also obtained from the mice immediately after WIR/sham stress to examine their functions. Neutrophil superoxide production was examined using 6 (2-h WIR), 11 (1-h WIR), and 19 (control) mice; neutrophil microsphere-phagocytosis was examined using 24, 21, and 28 mice, respectively; bactericidal activity of neutrophils was examined using 11, 11, and 12 mice, respectively; and plasma cortisol levels and neutrophil counts were examined using 21 (13 for neutrophil count), 11, and 18 (only for plasma cortisol) mice, respectively (summarized in the Supplemental Table).

### Treadmill exercise

Mice were exercised on an airtight murine treadmill (MK-680AT02; Muromachi Co., Tokyo, Japan), and their respiratory gases were measured using an O_2_/CO_2_ metabolism measuring system (MK-5000RQ; Muromachi Co.). Electric stimulation (50 volts) was used to encourage the mice to run. Treadmill exercise was started at 10 m/min with a 5° slope and was increased by 5 m/min every 3 min up to a maximum speed of 30 m/min. The murine treadmill exercise lasted until exhaustion (or 130 min in the control mice). When the mice remained at electric grids placed at the end of the treadmill for 2 seconds, we stopped the electric current of the grid and encouraged mice to run by tapping the treadmill box. When the mice repeated riding on the grid three times, we defined the mice as exhausted and stopped their treadmill exercise. We ran 50 control mice to obtain robust baseline data on treadmill exercise, and also ran 15 mice immediately after 2-h WIR (2-h WIR group) and 10 mice after 1-h WIR (1-h WIR group). Using these running mice, we measured their O_2_ consumption and CO_2_ production, as described below.

### Metabolic and respiratory measurements

O_2_ consumption and CO_2_ production were monitored during the treadmill exercise using an airtight treadmill and an O_2_/CO_2_ metabolism measuring system. The RER was calculated as the ratio of CO_2_ production over O_2_ consumption (CO_2_ production/O_2_ consumption). EE was calculated using the Weir equation (kcal/min = 3.941 × O_2_ consumption + 1.106 × CO_2_ production), as previously described^36–38^. Total EE was obtained from the integrated EE until exhaustion.

### Isolation of neutrophils

As described previously^12,26,39^, blood samples were withdrawn into a heparinized syringe from the abdominal inferior vena cava under lethal anesthesia with isoflurane (Forane^®^; AbbVie GK). In brief, leukocytes were isolated by dextran sedimentation. Blood samples were gently mixed with 6% dextran solution (Sigma-Aldrich, Deisenhofen, Germany) at a 4:1 volume ratio and held vertically for 15 min. After obtaining the supernatant, neutrophils were separated from mononuclear cells by centrifugation using Pancoll for mouse (PAN Biotech GmbH, Aidenbach, Germany) followed by hypotonic lysis of erythrocytes. The resultant cells contained nearly 90% neutrophils, as assessed by microscopy with Wright-Giemsa stain (Wako Pure Chemical Industries, Ltd. Osaka, Japan).

### Determination of the production of superoxide by neutrophils

The production of superoxide by neutrophils was determined by MCLA-dependent chemiluminescence as described previously^26,39^. A cuvette containing neutrophils (5 × 10^5^ cells) and MCLA (2 μM) (Tokyo Kasei Kogyo Co., Tokyo, Japan) in 200 μL of Hank’s Balanced Salt Solution (HBSS; Thermo Fisher Scientific K.K., Tokyo, Japan) was placed in a luminometer (Gene Light GL-200; Microtec Co., Chiba, Japan). Thereafter, 4 μg of phorbol 12-myristate 13-acetate (PMA; Sigma-Aldrich) was added to the cuvette to determine the increase in MCLA-dependent chemiluminescence intensity. After adding SOD (final concentration: 0.5 μM; Sigma-Aldrich), the superoxide production was determined as a decrease in chemiluminescence intensity by SOD^26,39^. We determine the neutrophil superoxide production in 6 mice in the 2-h WIR group, 11 mice in the 1-h WIR group, and 19 mice in the control group.

### Determination of the microsphere-phagocytosis by neutrophils

Neutrophils (5 × 10^5^ cells) were incubated with Fluoresbrite YG (=FITC) Carboxylate Microspheres (75 nm diameter; Polysciences Europe, Eppelheim, Germany; hereafter referred to as FITC-microspheres, 1 × 10^8^/mL) in 200 μL of 10% fetal bovine serum (FBS)-Roswell Park Memorial Institute (RPMI)-1640 medium (Sigma-Aldrich) for 20 min in 5% CO_2_ at 37 °C. After staining neutrophils with phycoerythrin (PE)-conjugated anti-mouse Gr-1 mAb (*e*Bioscience, San Diego, CA, USA), the phagocytosis of the FITC-microspheres by Gr-1^+^ neutrophils was analyzed using an FC500 instrument (Beckman Coulter Inc. Miami, FL, USA) as previously described^26,39^. The FITC fluorescent intensity is dependent on the number of ingested microspheres. Peaks on the histogram correspond to neutrophils that contain no ingested microspheres or one (peak 1), two (peak 2), or more (peak ≥3) microspheres, from left to right, respectively (see Fig. 5A). We investigated the phagocytosis by neutrophils in 24 mice in the 2-h WIR group, 21 mice in the 1-h WIR group, and 28 mice in the control group.

### Evaluation of the bactericidal activity of neutrophils

Obtained neutrophils (5 × 10^5^ cells) were co-cultured with 1 × 10^5^ CFU of *Escherichia coli* (*E. coli*) (strain B, ATCC 11303; Sigma-Aldrich) for 6 h. Thereafter, the suspension was serially diluted with PBS, placed on agar plates, and incubated for 18 h to count viable bacteria, as described previously^26,39^. We examined the neutrophil bactericidal activity in 11 mice in both the 2-h and 1-h WIR groups, and 12 mice in the control group.

### Measurement of the neutrophil count and plasma cortisol level

Blood samples were obtained by retro-orbital sinus puncture from the mice under isoflurane anesthesia using heparinized Hematocrit Tubes (Hemato-Clad^®^; Drummond Scientific Co., Broomall, PA, USA) before WIR (10 μL) and after WIR (100 μL). Neutrophils obtained before and after WIR were promptly counted using an automatic blood cell counter for animals (PCE-210 N, Erma Inc., Tokyo, Japan). Plasma was obtained from the blood sample after WIR by centrifugation and stored at −80 °C until the assay. Plasma cortisol levels were measured by a mouse cortisol EIA kit (DetectX Kits; Arbor Assays, Ann Arbor, MI, USA). We measured the neutrophil counts and cortisol levels in 21 mice in the 2-h WIR group, 11 mice in the 1-h WIR group, and 18 mice in the control group.

### Statistical analyses

The data are presented as the means ± standard error (SE). Statistical analyses were performed using the StatView 4.02J software package (Abacus Concepts, Berkeley, CA). Statistical evaluations were performed using a standard one-way ANOVA, followed by the Bonferroni post-hoc test. Student’s *t*-test was employed to compare the data of two different groups. *P* values of < 0.05 were considered to indicate significant difference.

## Supplementary information

Supplemental table
