# Supplementary material for: The reduced bactericidal activity of neutrophils as an incisive indicator of water-immersion restraint stress and impaired exercise performance in mice
Source: Sci Rep. 2019 Mar 14;9:4562. doi: 10.1038/s41598-019-41077-5 (PMC6418158; doi:10.1038/s41598-019-41077-5)
Supplement: Supplementary file 1 — Supplemental table [file 41598_2019_41077_MOESM1_ESM.pdf]

Supplementary information

The reduced bactericidal activity of neutrophils as an incisive indicator of water-immersion restraint stress and impaired exercise performance in mice

Manabu Kinoshita[1], Hiroyuki Nakashima[1], Masahiro Nakashima[1], Minori Koga[2], Hiroyuki Toda[2], Kazuki Koiwai[1], Yuji Morimoto[3], Hiromi Miyazaki[4], Daizoh Saitoh[4], Hiroaki Suzuki[5], Shuhji Seki[1]

[1] Department of Immunology and Microbiology, National Defense Medical College, Namiki 3-2, Tokorozawa 359-8513, Japan

[2] Department of Psychiatry, National Defense Medical College, Namiki 3-2, Tokorozawa 359-8513, Japan

[3] Department of Physiology, National Defense Medical College, Namiki 3-2, Tokorozawa 359-8513, Japan

[4] Division of Traumatology, Research Institute, National Defense Medical College, 3-2 Namiki, Tokorozawa 359-8513, Japan

[5] Graduate School of Pure and Applied Sciences, University of Tsukuba, 1-1-1 Tennodai, Tsukuba, Ibaraki 305-8573, Japan

Suppl. Table The number of mice examined

Analytic item	Control	1-h WIR	2-h WIR	total
Treadmill running (analyzing exercise performance, O_2 consumption, and CO_2 production)	50	10	15	
Neutrophil superoxide production	19	11	6	
Neutrophil microsphere-phagocytosis	28	21	24	
Neutrophil bactericidal activity	12	11	11	
Measuring plasma cortisol	18*	11	21 (13) †	
Total	127	64	77	268

*only for measuring plasma cortisol levels, †13 mice for neutrophil count
